# Supplementary material for: Splice-Junction-Based Mapping of Alternative Isoforms in the Human Proteome
Source: Cell Rep. Author manuscript; Available in PMC 2020 Jan 15. (PMC6961840; doi:10.1016/j.celrep.2019.11.026)
Supplement: 2 [file NIHMS1546469-supplement-2.zip › DF1/PXD006675/RightAtrium/RightAtrium_48_MAP4_DSYVPLELAK.pdf]

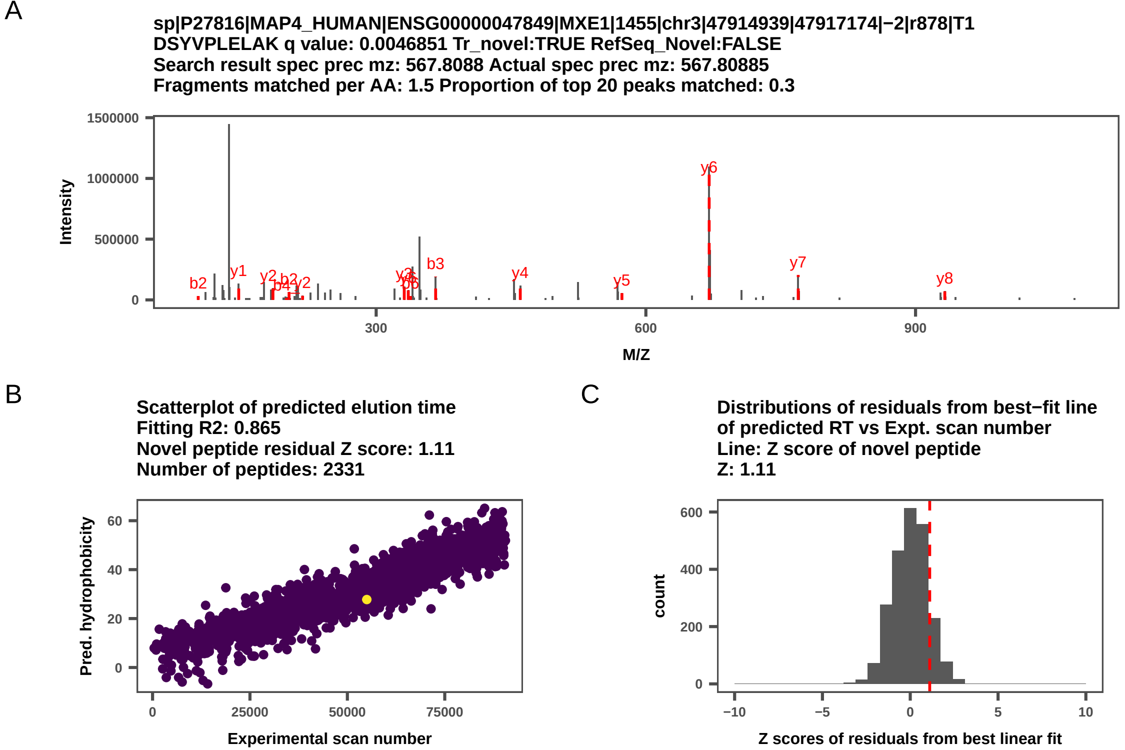
A
sp|P27816|MAP4_HUMAN|ENSG00000047849|MXE1|1455|chr3|47914939|47917174|−2|r878|T1
DSYVPLELAK q value: 0.0046851 Tr_novel:TRUE RefSeq_Novel:FALSE
Search result spec prec mz: 567.8088 Actual spec prec mz: 567.80885
Fragments matched per AA: 1.5 Proportion of top 20 peaks matched: 0.3
Intensity
1500000
1000000
500000
0
b2
y1
y2
b3
y4
y5
y6
y7
y8
300
600
900
M/Z
B
Scatterplot of predicted elution time
Fitting R2: 0.865
Novel peptide residual Z score: 1.11
Number of peptides: 2331
Pred. hydrophobicity
60
40
20
0
25000
50000
75000
Experimental scan number
C
Distributions of residuals from best−fit line
of predicted RT vs Expt. scan number
Line: Z score of novel peptide
Z: 1.11
count
600
400
200
−10
−5
5
10
Z scores of residuals from best linear fit
